# Sweet Memories or Not? A Comparative Study on Cognitive Impairment in Diabetes Mellitus

**DOI:** 10.3389/fpubh.2022.822062

**Published:** 2022-02-04

**Authors:** Sangeetha Merrin Varghese, Niva Joy, Anulekha Mary John, Geomcy George, George Mateethra Chandy, Anoop Ivan Benjamin

**Affiliations:** ^1^Department of Community Medicine, Believers Church Medical College, Thiruvalla, India; ^2^Medical Student, Believers Church Medical College, Thiruvalla, India; ^3^Department of Medicine, Believers Church Medical College, Thiruvalla, India; ^4^Department of Radiation Oncology, Believers Church Medical College, Thiruvalla, India; ^5^Department of Gastroenterology, Believers Church Medical College, Thiruvalla, India

**Keywords:** diabetes, cognitive impairment, ACE-3, cognitive dysfunction, high blood glucose

## Abstract

**Introduction:**

Type 2 Diabetes Mellitus is a modern-day epidemic and dementia has been declared as a global challenge. It is, therefore, worthwhile to investigate the effect that Diabetes has on cognition. Although effective screening is routinely carried out for various complications of Diabetes, its effect on Higher Mental Functions is often overlooked.

**Methodology:**

A cross-sectional analytical study to assess Cognitive Impairment was carried out on 800 participants, 400 diabetics and 400 non-diabetics attending a tertiary care center. The Addenbrooke's Cognitive Examination- III was used, which is a validated, highly sensitive tool having a maximum score of 100. Patients with a score < /= 82 were considered to have impaired Cognition. Statistical analysis was done using SPSSv.21. Suitable statistical tests like Mann–Whitney *U, t*-test, ROC curve and Logistic regression analysis were done.

**Results:**

Cognitive Impairment was present in 63.8% of the diabetics when compared to only 10.8% in the non-diabetics, with an Odds Ratio-8.78 (CI-4.47–17.22). The total ACE score in diabetics [median-82 (IQR-4), mean rank-270.06] was less compared to the non-diabetic patients [median- 85 (IQR-3), mean rank-530.94] (*U* = 27822, *p*-0.001). Attention, Memory, Language, and Visuospatial domains were significantly lower in the diabetics compared to the non-diabetics. However, the fluency domain was not affected. Hypertension and the presence of macrovascular diseases were significantly associated with Cognitive Impairment (*p* < 0.005). Those with Cognitive dysfunction also had higher mean RBS values and longer duration of Diabetes (*p*-0.001). The cut-off value for RBS (to distinguish people with and without Cognitive Impairment) from ROC curve was 142.5 (AUC = 0.834, Youden's Index-0.586, *p*-0.001) and for duration of Diabetes was 7.5 years (AUC = 0.847, Youden's Index-0.529, *p*-0.001).

**Conclusion:**

This paper highlights that Cognitive Impairment exists in a very high proportion of diabetic patients in Kerala. So, it is important that we do an early assessment of cognitive function in diabetes patients and manage them prudently. Early interventions may prove to be beneficial in the long run, considering the burden of diabetes and cognitive dysfunction associated with the disease.

## Introduction

Type-2 Diabetes Mellitus has become a modern day epidemic that is associated with significant mortality and morbidity. Kerala is taking the lead with a prevalence of more than 20% (1) and is named as the Diabetes Capital of India (2). Though various complications of diabetes are studied in-depth, its effects on higher mental functions (HMF) are often overlooked, due to lack of clear signs and unavailability of standard assessment techniques (3). Even mild forms of cognitive impairment may interfere with activities which require various cognitive domains such as general intelligence, processing speed, attention, perception, learning, memory, and executive function. Intact cognitive functions are an integral part and parcel of everyday activities related to remembering, making decision and solving problems or even personal issues and health issues. A meta-analysis showed small to moderate performance decline in each domain of a cognitive function in persons with diabetes relative to non-diabetic controls (4).

It has been estimated that an individual with diabetes mellitus is 1.5 times more likely to experience cognitive dysfunction and dementia than a normal healthy individual (5). Diabetes is associated with the development of cognitive impairment possibly because of its vascular and neurodegenerative effects through chronic hyperglycemia and hypoglycemia (5–7). In addition, insulin resistance, the major component of type 2 diabetes, may also be a risk factor for Alzheimer's Disease (AD) through increasing Abeta (beta-amyloid) generation in the brain (8). T2DM causes brain insulin resistance, oxidative stress, and cognitive impairment. A review even concluded that the term “type 3 diabetes” accurately reflected the fact that AD represents a form of diabetes that selectively involves the brain (9).

Dementia has been declared a global challenge, leading to loss of independence and non-adherence to medication, causing a great burden for the families of the patients. It also leads to enormous global annual costs, which are expected to increase significantly in the next few decades (10). Early detection of cognitive deterioration in the prodromal stage itself is arguably important in order to initiate preventive strategies in the future. Mild cognitive impairment (MCI) converts to dementia at a rate of ~10% per year (11). Alleviation of microvascular complications and hypoglycemia is the key in the treatment of DM to prevent cognitive decline. Recent studies suggest that certain interventions such as physical exercise can protect against dementia by exercise-induced synaptogenesis (12). Individuals who underwent exercise training showed modest improvements in attention, processing speed, executive function, and memory (13).

The Adenbrooke's Cognitive Examination-III (ACE-III) is a validated tool that can differentiate patients with and without cognitive impairment. It is sensitive to the early stages of dementia (14) and showed better sensitivity to detect dementia compared to the Mini -Mental State Examination (MMSE) (15). ACE-III more efficiently identifies everyday functional impairments compared with both MMSE and Montreal Cognitive Assessment (MoCA) (16), though the latter has been widely used to assess cognitive dysfunction.

The reason why it is important to study the cognition in Diabetes is that different behaviors and even clinical presentation will be affected by cognitive dysfunction, which can impact their self-care and the strategies to improve diabetes management. For example, a patient with executive dysfunction might be misconstrued as “non-compliance” when the changes recommended by the care providers are not actually integrated in to day-to day life. To our surprise, there are only a few studies on cognitive Impairment in Diabetes in Kerala, though it has the highest prevalence of Diabetes. So, this study was conducted to quantify the extent of cognitive dysfunction in diabetics when compared to non-diabetics and to explore the association between cognitive dysfunction and selected risk factors.

## Methodology

This is a cross-sectional analytical study conducted among diabetic and non-diabetic people between 41 and 60 years of age in a tertiary care center in Central Kerala during June- September 2021. The study was initiated after obtaining clearance from the Institutional Ethics Committee (IEC/2020/05/151). Informed consent was obtained from all participants and data was collected by interviewing the participants.

According to a study by Satyajeet Roy et al. on cognitive function and control of type 2 DM in adults, the prevalence of cognitive dysfunction was found to be 19.5% (17) and therefore, a total sample size of 800 was calculated to be sufficient (400 in diabetic and 400 in non-diabetic group).

### Inclusion Criteria

Only literate people between 40 and 60 years of age were included in the study so as to eliminate the possibility of senile dementia.

### Exclusion Criteria

Patients with known dementia due to any other cause, those with advanced co-morbid medical conditions and developmental disorders and those on drugs affecting cognitive functions such as sedatives, neuroleptics, antidepressants, anti-epileptics and anti- psychotic drugs, were excluded from the study.

We used Addenbrooke's cognitive examination III for assessing the cognitive function of the participants. It is a brief cognitive screening tool, which takes 20 min to administer. It is composed of tests of attention, memory, language, verbal fluency, and Visuo-spatial skills (14). The Malayalam translation of ACE-3 is used in the study which is a validated tool among the Malayalam-speaking population of South India (18). The total score of the ACE-3 is based on a maximum score of 100, with higher scores indicating better cognitive functioning. This tool was preferred over MMSE as it had higher sensitivity in detecting even milder forms/early stages of Cognitive Impairment (14). A score above 88/100 in ACE-3 is considered to be normal, a score between 83 and 88 is inconclusive and a score ≤ 82 shows Cognitive Impairment (19).

A digital BP apparatus and a glucometer were also used to assess the Blood pressure and Random Blood Glucose level of every participant.

The current WHO diagnostic criteria for diabetes, i.e., a person with fasting plasma glucose ≥7 mmol/l (126 mg/dl) or 2-h plasma glucose ≥11.1 mmol/l (200 mg/dl) or HbA1c ≥6.5 was considered to be diabetic.

Cognitive function can be defined as mental processes that lead to the gaining of knowledge and allows people to carry out their daily life activities. It includes a variety of mental processes such as perception, attention, memory, decision making, and language comprehension.

Hypertension is defined as a systolic BP ≥140 mmHg and Diastolic BP ≥90 mmHg in sitting position. Systolic blood pressure (SBP) and diastolic blood pressure will be noted down as a mean of two tests conducted after an interval of 15 min in sitting position.

Data analysis was done in SPSS v.21. Continuous variables were summarized using mean (SD) and median (IQR). Categorical variables were expressed using counts (%). Testing for significance was done by Chi-square test for categorical variables and independent sample *t*-test or Mann–Whitney (M–W) test for continuous variables. Logistic regression analysis was done to adjust for potential confounders. While using the M–W test, if the histogram of the cognitive score was different in the diabetic and non-diabetic groups, differences in mean ranks were summarized, rather than the median. A ROC curve was also constructed to identify whether RBS values and duration of diabetes could distinguish people with and without Cognitive Impairment.

## Results

There were 800 participants in the study, 400 diabetic and 400 non-diabetic people. Among the study participants, 52.38% belonged to the 41–50 years age group and 47.62% belonged to the 51–60 years age group. Males and female representation was nearly equal (49.75 vs. 50.25). Among the diabetics, 56% reported having subjective perception of a decline in cognitive function, compared to only 8% in the non-diabetics. A vast majority (nearly 99%) expressed their willingness to accept our recommendations to improve their cognitive function, if they were found to have cognitive deficits (see Table 1).

**Table 1:** Characteristics of study population.

**Factors**	**Diabetics (*n* =400)**	**Non-diabetics(*n* = 400)**
**Age**		
41–50	107 (26.8%)	312 (78%)
51–60	293 (73.3%)	88 (22%)
***Gender***		
Male	171 (42.8%)	227 (56.8%)
Female	229 (57.3%)	173 (43.3%)
**Subjective decline in-cognitive function**		
Yes	224 (56%)	32 (8%)
No	176 (44%)	368 (92%)
**Willingness to accept our recommendation to improve cognition**		
Yes	395 (98.8%)	396 (99%)
No	5 (1.3%)	4 (1%)

The presence of Cognitive Impairment was 63.8% among the diabetic patients when compared to only 10.8% among the non-diabetics (see Table 2). The odds of having Cognitive Impairment is 8.78 (CI-4.47–17.22) times higher in Diabetics when compared to the non-diabetic population.

**Table 2:** Factors associated with cognitive impairment.

**Factors**	**Cognitive impairment present**	**No cognitive impairment**	**Total**	**OR**	**C.I. (OR)**	***p*-value**
Diabetic status						
Diabetic	255 (63.8%)	145 (36.2%)	400	8.78	4.47–17.22	0.001
Non-diabetic	43 (10.8%)	357 (89.2%)	400			
HTN						
Present	114 (67.1%)	56 (32.9%)	170	2.945	1.218–7.12	0.016
Absent	184 (29.2%)	446 (70.8%)	630			
Smoking						
Present	62 (52.5%)	56 (47.5%)	118	1.614	0.758–3.435	0.214
Absent	236 (34.6%)	446 (65.4%)	682			
Alcoholism						
Present	82 (40%)	123 (60%)	205	1.144	0.567–2.311	0.707
Absent	216 (36.3%)	379 (63.7%)	595			
H/o macrovascular disease						
Present	20 (95.2%)	1 (4.8%)	21	11.905	1.309–108.28	0.028
Absent	278 (35.7%)	501 (64.3%)	779			
Gender						
Male	133 (33.4%)	265 (66.6%)	398	0.951	0.531–1.705	0.866
Female	165 (41%)	237(59%)	402			
Age						
40–50 years	32 (7.6%)	387 (92.4%)	419	0.075	0.045–0.127	0.001
51–60 years	266 (69.8%)	115 (30.2%)	381			

**Figure 1:**
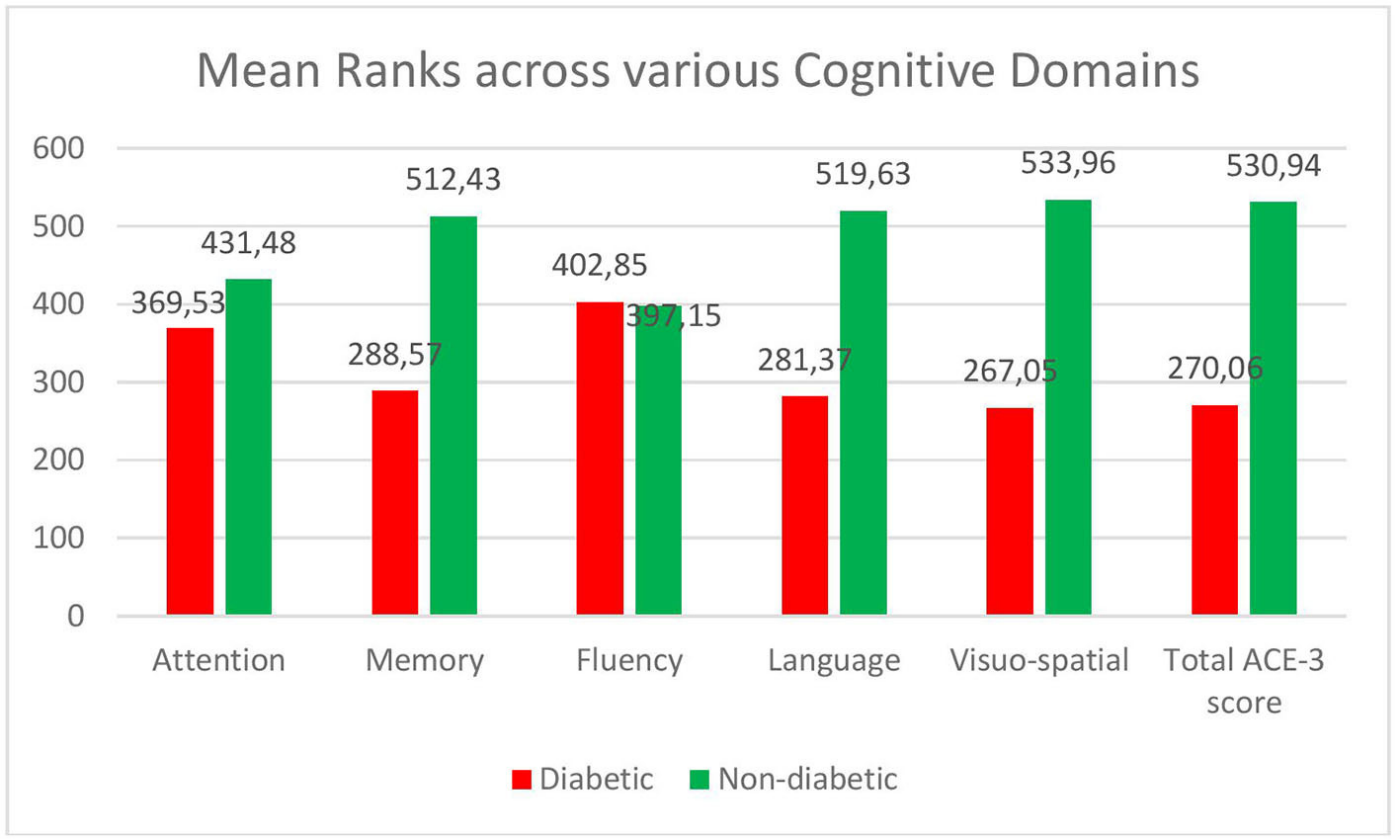
Comparison of various cognitive domains across diabetics and non-diabetics.

The total ACE score in diabetics [median-82 (IQR-4), mean rank-270.06] was less compared to the non-diabetic patients [median-85 (IQR-3), mean rank-530.94] (see Figure 1). The difference was statistically significant on the Mann–Whitney *U* test (*U* = 27,822, *p*-0.001). The mean ranks across the various cognitive domains—Attention, Memory, Language, and Visuospatial domains were significantly lower in the diabetics compared to the non-diabetics. However, the fluency domain was not affected in Diabetes (see Figure 1).

Hypertension and history of macrovascular diseases like Myocardial Infarction and cerebrovascular accidents were significantly associated with Cognitive Impairment (*p* < 0.005). The mean systolic Blood pressure was higher in those with cognitive impairment compared to those without impairment in cognition [134.07 (19.11) vs. 122.88 (15.33)] (see Table 2).

The mean RBS values were significantly higher among those with Cognitive Impairment compared to those without cognitive impairment [193.91 (59.42) vs. 134.02 (35.44), *p* = 0.001). The duration of diabetes played a significant role in Cognition with the mean duration of diabetes being much higher among those with cognitive impairment [8.98 yrs (2.94) vs. 5.32 yrs (2.54), *p* = 0.001) (see Table 3). Those in the age group of 51–60 years had more cognitive impairment when compared to the age group 41–50 years. Although a gender difference was also observed in the propensity to have cognitive impairment, with females more likely to have a cognitive impairment when compared to males, it was not statistically significant. However, the mean RBS values were also significantly higher in females when compared to males [163.26 (57.69) vs. 149.33 (49.56), *p* = 0.001).

**Table 3:** Comparison of duration of diabetes, mean RBS and systolic blood pressure across varying levels of cognition.

	**Cognitive impairment present**	**No cognitive impairment**	***T*-value, *p*-value**
RBS (mean, sd)	193.91 (59.42)	134.02 (35.44)	15.80, 0.001
Systolic blood pressure (mean, sd)	134.07 (19.11)	122.88 (15.33)	8.60, 0.001
Duration of diabetes in **years** (mean, sd)	8.98 (2.94)	5.32 (2.54)	12.56, 0.001

In multivariate Logistic regression model, Diabetic status, higher age group, high blood sugar level, systolic hypertension, and presence of macrovascular diseases emerged as independent predictors of Cognitive Impairment (see Table 2).

**Figure 2:**
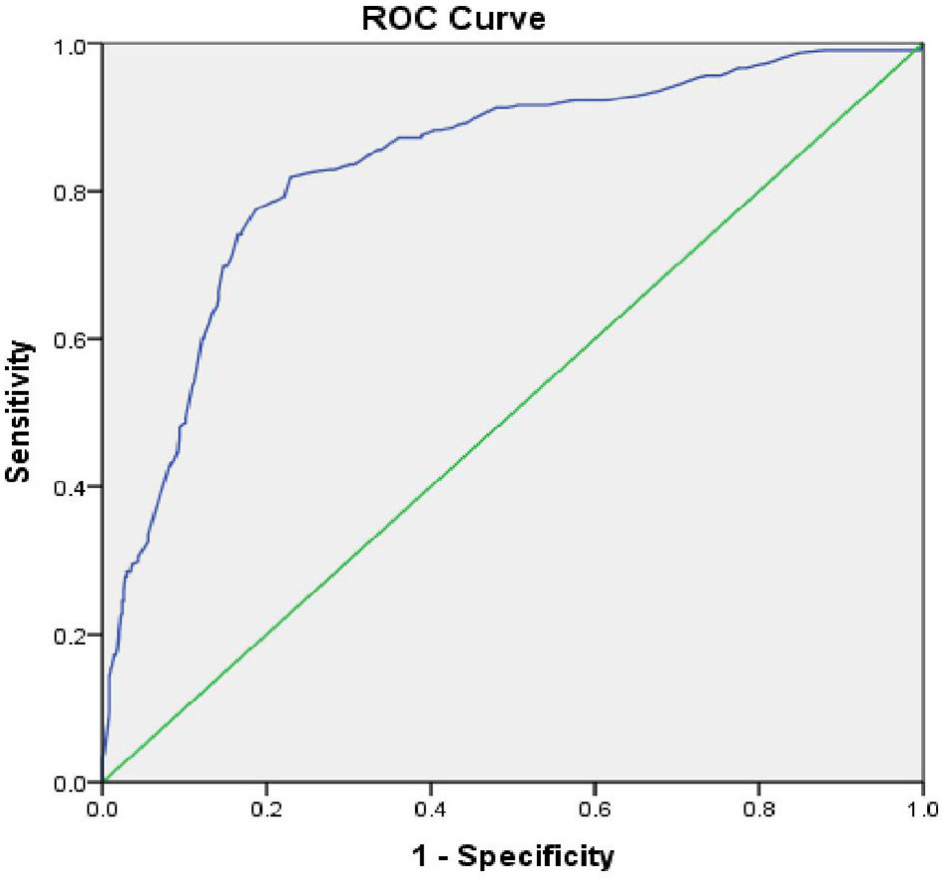
ROC curve––random blood sugar values with cognitive impairment.

The Area under the Curve (AUC) of the ROC curve constructed using Random Blood Sugar Values was 0.834 and *p* = 0.001. Therefore, RBS values are useful to distinguish people with and without Cognitive Impairment (see Figure 2). The cut-off for RBS was 142.5, based on the maximum calculated Youden's Index = 0.546, Sensitivity-82.9%, Specificity-71.7%.

**Figure 3:**
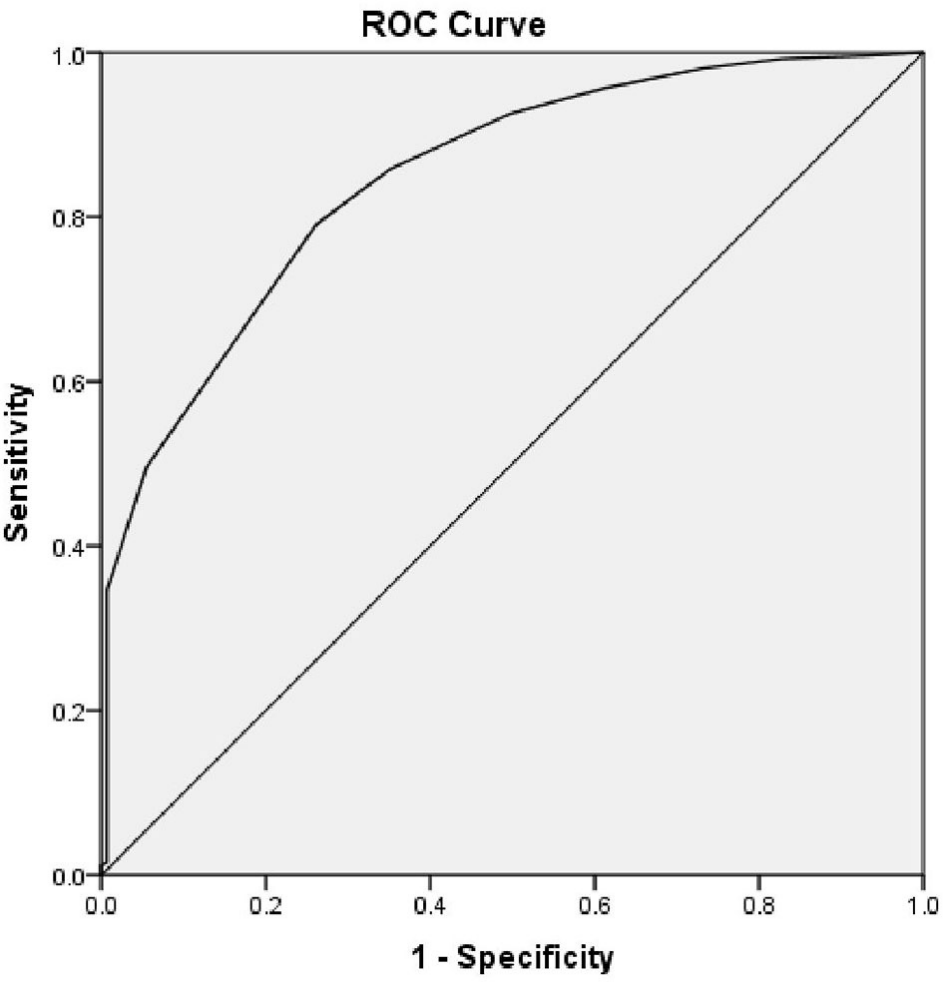
ROC curve—duration of diabetes with cognitive impairment.

Similarly, the AUC of the ROC curve for Duration of Diabetes and Cognitive Impairment was 0.847 and *p* = 0.001. Therefore, the duration of diabetes is also a useful indicator to detect Cognitive Impairment with a sensitivity of 79.1% and specificity of 73.8%. The cut-off for the duration of Diabetes was 7.5 years, based on the maximum calculated Youden's Index = 0.529 (see Figure 3).

## Discussion

Evidence suggests that diabetes predisposes to cognitive decline and Type-2 Diabetes Mellitus is associated with a 50 % increase in the risk for dementia (20). It has been associated with impaired attention, processing and motor speed, executive functioning, and verbal memory (21). In comparison to those without diabetes, diabetes in midlife is associated with a 19 % greater cognitive decline over 20 years (22). It is important to quantify the extent of cognitive impairment because of the increasing prevalence of T2DM and prolonged life span, so that preventive strategies can be initiated early in life. Also, lifestyle factors such as cigarette smoking and obesity also predispose to dementia (23). Lifestyle interventions that couple aerobic exercise with tailored dietary interventions currently offer the most likely benefit (24).

Patients who have metabolic syndrome are also at increased risk of developing cognitive dysfunction. Metabolic disorders like hyper or hypoglycemia, polyol pathway and accumulation of advanced glycation end products are already well-recognized in the pathophysiology of neurocognitive disorders like Alzheimer's disease (25). Hyperglycemia was the main contributor to the observed association of metabolic syndrome and cognition (26). This study brings to light the impact of hyperglycemia on cognitive function and helps to identify even mild impairment in cognition, which may be subtle to start with and often goes unrecognized by both patients and clinicians.

Our study reveals that Cognitive Impairment was 63.8% among people with diabetes when compared to 10.8% in the normal population, with an Odds Ratio of 5.93. ACE score was lesser in diabetics and presence of Hypertension or a previous history of Myocardial Infarction/ Cerebrovascular event further increased the risk of cognitive dysfunction. Also, diabetes had affected four out of five cognitive sub-domains tested, which includes Attention, Memory, Language, and Visuo-spatial ability. This is important, as cognitive dysfunction in various domains affect behaviors such as judgment, problem solving, starting new behaviors, or stopping old behaviors. This may impact a person's ability to manage his or her diabetes as it affects one's self care, problem solving skills, motivation to follow instructions, and adhere to exercise regimens. Hence impaired cognition can by itself lead to a vicious cycle of uncontrolled sugars and further cognitive dysfunction. A similar study was conducted in Kerala using Montreal Cognitive Assessment and cognitive impairment among the diabetics was found to be 54.29% (27). However, as mentioned earlier, ACE- III being more sensitive to detect dementia when compared to MMSE and MoCA, our results align with their findings and highlight the fact that a significant proportion of people with diabetes have at least mild cognitive impairment when compared to non-diabetics after adjusting for age, gender and other risk factors.

In our study, the mean RBS values were significantly high among those who had Cognitive Impairment. Also, RBS values could reliably distinguish people with Cognitive impairment (AUC = 0.834 and *p* = 0.001). Other studies also suggest that severity of diabetes is a risk factor for developing dementia (28). To make matters more worrying, it is found that individuals without diabetes who have higher average glucose levels also have a significant risk for dementia (29). The adjusted hazard ratio for dementia in people with a minimally increased glucose level of 6.4 mmol/L (115 mg/dl) was 1.18 (1.04–1.33), suggesting that elevated serum glucose level may be an independent risk factor for cognitive dysfunction (29). Hyperglycemia-mediated advanced glycosylated end product production and oxidative stresses are cited as the factors that can damage neurons and vascular endothelium leading to cognitive dysfunction (30).

A recent pooled analysis of 14 studies examined data from 2.3 million individuals and over 100,000 incident cases of dementia from cohorts from Asia, Europe, and the Americas and found that diabetes was associated with 60% increased risk of dementia and 18% excess risk in women (31). In our study, females were found to have higher blood sugar levels when compared to males, which could be the reason for posing an increased risk for dementia in women.

The duration of Diabetes was also an important contributor to cognitive dysfunction. Cognitive decline is more prominent when the duration of Diabetes is more than 5 years and hypertension further accelerates the risk (32). This was consistent with our study findings and the cut-off for the presence of cognitive dysfunction was calculated as 7.5 years of duration of Diabetes. A study of a middle-aged population showed that tight glycemic control during midlife may protect against cognitive decline in later life. There is also clear evidence that intensive control of blood glucose and blood pressure is not beneficial in preventing cognitive decline in later life. So, it is important that we do an early assessment of cognitive function in diabetes patients and manage them prudently.

Early identification of Diabetes as well as Cognitive Dysfunction is essential for protecting against cognitive decline in later life by early introduction of aerobic exercises, cognitive exercises and lifestyle modification.

## Conclusion

This paper highlights that Cognitive Impairment exists in a very high proportion of diabetic patients in Kerala. High blood sugar levels, longer duration of Diabetes, hypertension and presence of a Cerebrovascular event or Myocardial Infarction were associated with a higher risk of cognitive decline. Assessment of cognitive function needs to be included in the clinical assessment of diabetics initially as well as on periodic follow-up, as it impacts the quality of life and self-care in people suffering from deficits in cognition. Also, the people expressed their willingness to accept measures which would be beneficial in preventing cognitive decline. Hence, early interventions may prove to be beneficial in the long run, considering the burden of diabetes and cognitive dysfunction associated with the disease.

## Data Availability Statement

The raw data supporting the conclusions of this article will be made available by the authors, without undue reservation.

## Ethics Statement

The studies involving human participants were reviewed and approved by Institutional Ethics Committee, Believers Church Medical College, Kerala. Written informed consent for participation was not required for this study in accordance with the national legislation and the institutional requirements.

## Author Contributions

SV contributed to the conception or design of the work, analysis, or interpretation of data for the work, and drafting the work. NJ contributed to conception and data collection. NJ, AJ, GC, GG, and AB revising it critically for important intellectual content and final approval of the version to be published. All authors contributed to the article and approved the submitted version.

## Conflict of Interest

The authors declare that the research was conducted in the absence of any commercial or financial relationships that could be construed as a potential conflict of interest.

## Publisher's Note

All claims expressed in this article are solely those of the authors and do not necessarily represent those of their affiliated organizations, or those of the publisher, the editors and the reviewers. Any product that may be evaluated in this article, or claim that may be made by its manufacturer, is not guaranteed or endorsed by the publisher.
